# Low serum level of miR-485-3p predicts poor survival in patients with glioblastoma

**DOI:** 10.1371/journal.pone.0184969

**Published:** 2017-09-20

**Authors:** Zhi-Qiang Wang, Mei-Yin Zhang, Mei-Ling Deng, Nuo-Qing Weng, Hui-Yun Wang, Shao-Xiong Wu

**Affiliations:** 1 Department of Radiation Oncology, Sun Yat-Sen University Cancer Center, Guangzhou, China; 2 State Key Laboratory of Oncology in South China, Sun Yat-Sen University Cancer Center, Guangzhou, China; 3 Collaborative Innovation Center for Cancer Medicine, Guangzhou, China; Universidad de Navarra, SPAIN

## Abstract

MicroRNAs (miRNAs) are short noncoding RNAs that play critical roles in human malignancies and can be used as biomarkers for cancer. Until now, a number of biomarkers for prognosis of glioblastoma (GBM) have been reported in tumor tissues but only a few biomarkers in circulating fluid. Using a custom microarray, we previously identified 19 differentially expressed miRNAs in serum of patients with GBM. In this study, we investigated whether 3 of the 19 miRNAs in serum could be used as prognostic biomarkers for patients with GBM. We first validated the serum levels of 3 candidate miRNAs in an independent cohort of 24 GBM patients and 12 healthy volunteers by real-time quantitative reverse transcription PCR (qRT-PCR), and then evaluated the prognostic value of these miRNAs in a total of 36 GBM patients. The results show that the serum levels of the 3 miRNAs (miR-451a, miR-485-3p and miR-4298) determined by qRT-PCR are significantly different between 24 GBM patients and 12 healthy volunteers (all P <0.05) and are in concordance with the results of microarray analysis. High serum level of miR-451a is correlated with positive tumor O(6)-methylguanine-DNA methyltransferase (*MGMT*) expression (P = 0.040). Survival analysis showed that low serum miR-485-3p level is associated with poor progression-free survival (PFS) (*P* < 0.004) and overall survival (OS) (*P* < 0.023). Furthermore, univariate and multivariate Cox analyses demonstrated that that serum miR-485-3p expression is a significant independent prognostic factor for PFS and OS in GBM patients. In conclusion, serum miR-485-3p level is reduced and might be a potential prognostic biomarker in GBM patients.

## Introduction

Glioblastoma (GBM) is the most common malignant primary brain tumor in adults. Despite advances in treatment, patients with GBM have a very poor outcome with a 5-year overall survival rate of less than 10% [1, 2]. To improve the survival of GBM patients, it is critical to understand the molecular mechanism of GBM development and progression and identify the molecular biomarkers for prognosis and targeted therapy. At present, many molecular biomarkers for prognosis have been identified from GBM tissues, such as O-6-methylgua-nine-DNA methyltransferase (MGMT), isocitrate dehydrogenase 1 and 2 (IDH1/2), p53, epidermal growth factor receptor (EGFR), platelet-derived growth factor receptor (PDGFR), phosphatase and tensin homolog (PTEN), phosphoinositide 3-kinase (PI3K), and 1p/19q [3]. These biomarkers also might be used as targets of gene therapy. However, though some of these biomarkers, such as p53 [4] and IDH1 [5] can be detected in peripheral blood, most of these biomarkers only can be detected in tumor tissues from GBM patients with surgical resection. Therefore, easily accessible and non-invasive biomarkers, which can be detected in GBM patients with or without surgery, is critical to more effective management of GBM.

MicroRNAs (miRNAs) are small endogenous noncoding RNA molecules that play important roles in a variety of physiological and pathological processes including tumorigenesis and tumor progression [6–11]. Tumor specific circulating miRNAs were first detected in the serum of patients with diffuse large B-cell lymphoma in the year 2008 [12]. Recently, more and more studies suggested that circulating miRNAs can be very stable in biofluid and might be used as potential molecular biomarkers for human malignancies [13–15]. However, there are only a few studies on circulating miRNAs in serum, plasma or cerebrospinal fluid (CSF) of glioma patients, including GBM [16–18]. Therefore, identification of circulating miRNAs as biomarkers will be helpful for clinicians in the diagnosis, prognosis and treatment of GBM patients.

Using a custom microarray platform containing 1843 species of miRNAs, we previously identified 19 serum miRNAs with differential expression between 22 GBM patients and 8 healthy volunteers [19]. Here, we first validated expressions of 3 serum miRNAs of the 19 miRNAs in a cohort of 24 GBM patients and 12 healthy volunteers using real-time quantitative reverse transcription PCR (qRT-PCR), and then determined which serum miRNAs might be used as a molecular biomarker for predicting the prognosis in 36 GBM patients.

## Materials and methods

### Patients and clinical samples

Blood samples were obtained from 36 patients with GBM before surgery at the Sun Yat-Sen University Cancer Center between July 2011 and April 2014. The patients meeting the following inclusion and exclusion criteria were included in this study: (1) macroscopically complete resection of tumor; (2) no treatment before surgery; (3) first diagnosed as GBM by pathology after surgery; (4) receiving intensity-modulated radiotherapy plus concomitant temozolomide treatment with or without adjuvant temozolomide chemotherapy; (5) no other cancers at diagnosis. All patient characteristics are shown in Table 1. The patients included 21 males and 15 females. The median age of patients at the time of diagnosis was 48 years (range 16–76 years). None of the patients had received chemotherapy or radiotherapy before surgery. After surgery, all patients received radiotherapy combined with chemotherapy. The latest follow-up was updated in March 2015, and the median time of follow-up was 17.4 months (range 5.6–44.8 months). Overall survival (OS) is defined as the time from the date of surgery to the date of death from GBM or last date of follow-up; Progression-free survival (PFS) is defined as the time from the date of surgery to the date of relapse or metastasis of GBM or death from GBM or last date of follow-up. Control blood samples were collected from 12 healthy age- and sex-matched volunteers (median age: 48 years, age range 18–70 years). Blood samples (3 mL) were drained into tubes, and then centrifuged for 15 min at 1200g to separate buffy coats and serum. The serum was aspirated, aliquoted and stored at -80°C until use. This study was approved by the Research Ethics Committee of Sun Yat-Sen University Cancer Center. In this study, all participants or guardians on behalf of the participants whose age was under 18 years old signed a written informed consent form for the use of their blood samples before surgery.

**Table 1:** Correlation of serum levels of three miRNAs with clinicopathological factors in GBM patients.

Characteristic	Case No (%)	miR-451a Level			miR-4298 Level			miR-485-3p Level		
		High	Low	*P value*	High	Low	*P value*	High	Low	*P value*
Gender										
Male	21 (58.3)	10 (47.6)	11 (52.4)	0.735	13 (61.9)	8 (38.1)	0.091	9 (42.8)	12 (57.2)	0.500
Female	15 (41.7)	8 (53.3)	7 (46.7)		5 (33.3)	10 (66.7)		9 (60.0)	6 (40.0)	
Age (years)										
< 45	14 (38.9)	9 (64.3)	5 (35.7)	0.171	5 (35.7)	9 (64.3)	0.171	6 (42.9)	8 (57.1)	0.733
≥ 45	22 (61.1)	9 (40.9)	13 (59.1)		13 (59.1)	9 (40.9)		12 (54.5)	10 (45.5)	
Preoperative KPS										
≥ 80	25 (69.4)	14 (56.0)	11 (44.0)	0.278	11 (44.0)	14 (56.0)	0.278	13 (52.0)	12 (48.0)	1.000
< 80	11 (30.6)	4 (36.4)	7 (63.6)		7 (63.6)	4 (36.4)		5 (45.5)	6 (54.5)	
Surgery procedure										
GTR	14 (38.9)	5 (35.7)	9 (64.3)	0.171	7 (50.0)	7 (50.0)	1.000	6 (42.9)	8 (57.1)	0.733
PR	22 (61.1)	13 (59.1)	9 (40.9)		11 (50.0)	11 (50.0)		12 (54.5)	10 (45.5)	
Tumor size										
< 5 cm	16 (44.4)	6 (37.5)	10 (62.5)	0.180	6 (37.5)	10 (62.5)	0.180	10 (62.5)	6 (37.5)	0.315
≥ 5 cm	20 (55.6)	12 (60.0)	8 (40.0)		12 (60.0)	8 (40.0)		8 (40.0)	12 (60.0)	
Tumor MGMT expression										
Negative	14 (38.9)	4 (28.6)	10 (72.4)	0.040	8 (57.1)	6 (42.9)	0.494	8 (57.1)	6 (42.9)	0.733
Positive	22 (61.1)	14 (63.6)	8 (36.4)		10 (45.5)	12 (54.5)		10 (45.5)	12 (54.5)	

Abbreviations: KPS, Karnofsky Performance Scale; MGMT, O(6)-methylguanine-DNA methyltransferase detected by immunohistochemistry; GTR, gross total resection; PR, partial resection.

### MicroRNA isolation

RNA was isolated from serum samples using the Plasma/Serum Circulating and Exosomal RNA Purification Mini Kit (Slurry Format) (Norgen Biotek Corporation, Thorold, Ontario, Canada, L2V4Y6) according to manufacturer’s instructions. Briefly, 0.3 mL of Slurry C2 solution and 2.7 mL of Lysis Buffer A were added to 1.5 mL of serum and mixed well. The mixture was incubated for 10 minutes at 60°C. After incubation, 4.5 mL of 100% ethanol was added into the tubes and mixed well. The tubes were centrifuged for 30 seconds at 201 g (1000 RPM), and then the supernatants were discarded. Lysis buffer A (0.45 mL) was added into the tubes again to dissolve the slurry pellet and incubated for another 10 minutes at 60°C. After adding 0.45 mL of ethanol, the mixtures were loaded onto the provided column and centrifuged for 1 minute at 20871 g (14000 RPM). After discarding the eluate and washing the column with 400 μL of Wash Solution A for 3 times, 100 μL of Elution Solution A was applied to the column, centrifuged and collected the eluate. The concentration and purity of Eluted RNA were determined by NanoDrop 1000 (Thermo Fisher Scientific, Wilmington, DE). The yields of total eluted RNA were 140–360 ng per 1500μL of serum.

### Real-time quantitative reverse transcription-PCR

The isolated serum RNA was first reverse transcribed using the TaqMan miRNA reverse transcription kit (Applied Biosystems) according to the instruction of the manufacturer. The quantitative PCR was performed using TaqMan microRNA assays (Applied Biosystems) in an ABI PRISM 7900 real-time PCR system. The primers of human miR-16, used as an endogenous control [20–23], were also purchased from Applied Biosystems. The assay ID number is 001141 for mir-451a, 001277 for mir-485-3p, 465290_mat for mir-4298, 000391 for mir-16. Reaction mixtures were incubated at 95°C for 10 minutes, followed by 40 cycles at 95°C for 15 seconds and 60°C for 1 minute. Signals were detected at the end of each cycle. The cycle threshold (Ct) values were calculated with SDS 2.4 software (Applied Biosystems). The assays were undertaken in triplicate for all samples. 2^–ΔCt^ represents the normalized miRNAs expression level.

### Statistical analyses

All statistical analyses were carried out using the software of SPSS 16.0 (SPSS Inc.). Data are expressed as mean ± SD. ANOVA was used to determine the statistical differences among groups. 2^–ΔCt^ was used to represent serum expression levels of miRNA in GBM patients and healthy volunteers detected by qRT-PCR. Unpaired t test was conducted to analyze the differential miRNA expression levels between tumors and normal controls. The correlation between two dichotomous variables was assessed using Chi-square test or Fisher's exact test. OS/PFS was calculated from the date of the initial surgery to the time of death. Survival curves were made by the Kaplan-Meier method, and the log-rank test was used to assess survival differences between the groups. Cox regression analysis was used to assess predictors related to survival. The significance level was set at P<0.05.

## Results

### Verification of the selected serum miRNA levels by qRT-PCR

We previously used a microarray-based technique to genome-widely analyze serum miRNA profile and identified 19 miRNAs that have significantly differential expression levels between 22 GBM patients and 8 normal volunteers [19], and the microarray data had been deposited into the GEO database in NCBI website with accession number GSE93850 (https://www.ncbi.nlm.nih.gov/geo/query/acc.cgi?acc=GSE93850). To verify the serum level of the miRNAs, we detected three of the 19 differentially expressed miRNAs using qRT-PCR in another 24 GBM patients and 12 matched healthy volunteers. Of the 3 detected miRNAs, miR-451a level was the highest and miR-4298 level was the lowest among the 19 differentially expressed miRNAs in our previous study. Another miRNA, miR-485-3p, was decreased in serum of GBM patients and also selected for validation based on the published data that showed its aberrant expression in human cancer cell lines [24–27].

**Fig 1:**
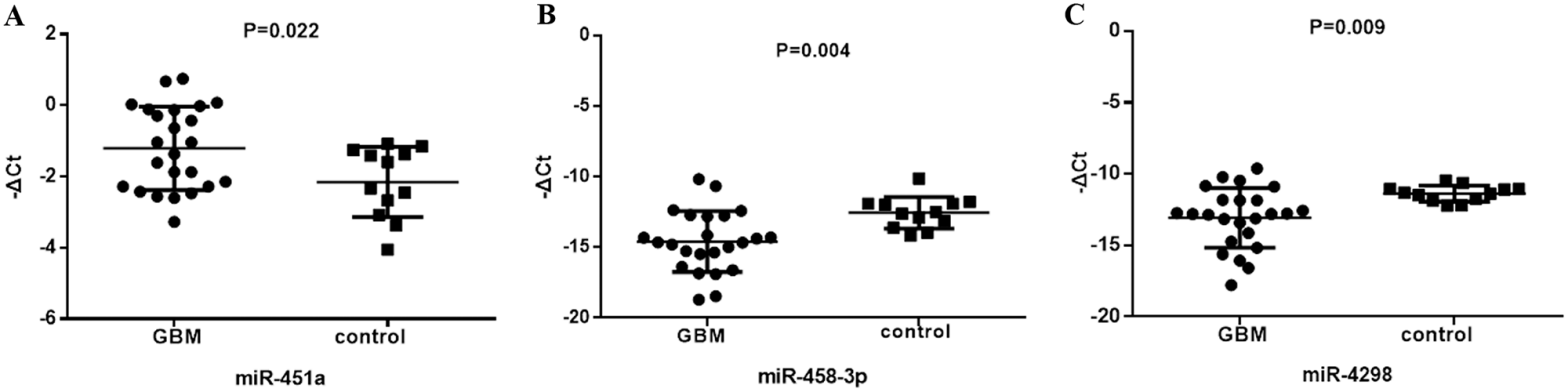
Validation of serum levels of miR-451a, miR-485-3p and miR-4298 by qRT-PCR in GBM patients. The average serum levels of the 3 miRNAs in GBM patients (n = 24) were compared with those in healthy volunteer controls (n = 12). **(A)** serum level of miR-451a in GBM patients is significantly higher than that in healthy controls. **(B)** serum level of miR-485-3p in GBM patients is significantly lower than that in healthy controls. **(C)** serum level of miR-4298 in GBM patients is significantly lower than that in healthy controls. The serum levels of the 3 miRNAs in GBM patients are in concordance with the results of microarray analysis. miRNA levels in each sample were normalized to miR-16. ΔCt = mean value of Ct (reference miRNA16)–mean value of Ct (target miRNA).

The relative expression of serum miR-451a, miR-485-3p and miR-4298 were depicted in the scatter plot of Fig 1. The results showed that the expression of the three miRNAs detected by qRT-PCR were significantly different between 24 GBM patients and 12 healthy volunteers (all P<0.05, Fig 1), of which 2 serum miRNAs (miR-485-3p, Fig 1B and miR-4298, Fig 1C) were significantly lower, while another serum miRNA (miR-451a, Fig 1C) was significantly higher in GBM patients than in healthy volunteers. The results are consistent with the microarray data, suggesting that microarray data is reliable and reproducible.

### Correlation of serum miRNAs with clinicopathological factors of GBM patients

We next examined the expression levels of the 3 miRNAs in another 12 patients with GBM by qRT-PCR. To further investigate the roles of serum miR-485-3p expression levels in GBM progression, the relationship between the 3 serum miRNAs expression levels and clinicopathological features was statistically analyzed in all 36 GBM patients. Patients were classified into a low- or high-expression group according to their miRNA expression levels used the median expression level of miRNAs as the cutoff point. Statistical analysis shows that high miR-451a level is associated with positive MGMT expression in tumor (detected by immunohistochemistry) (P = 0.040, Table 1), and no significant relationship has been observed between the 3 serum miRNAs expression and other clinicopathological features (P > 0.05, Table 1).

### Serum miR-485-3p is a prognostic marker in patients with GBM

**Fig 2:**
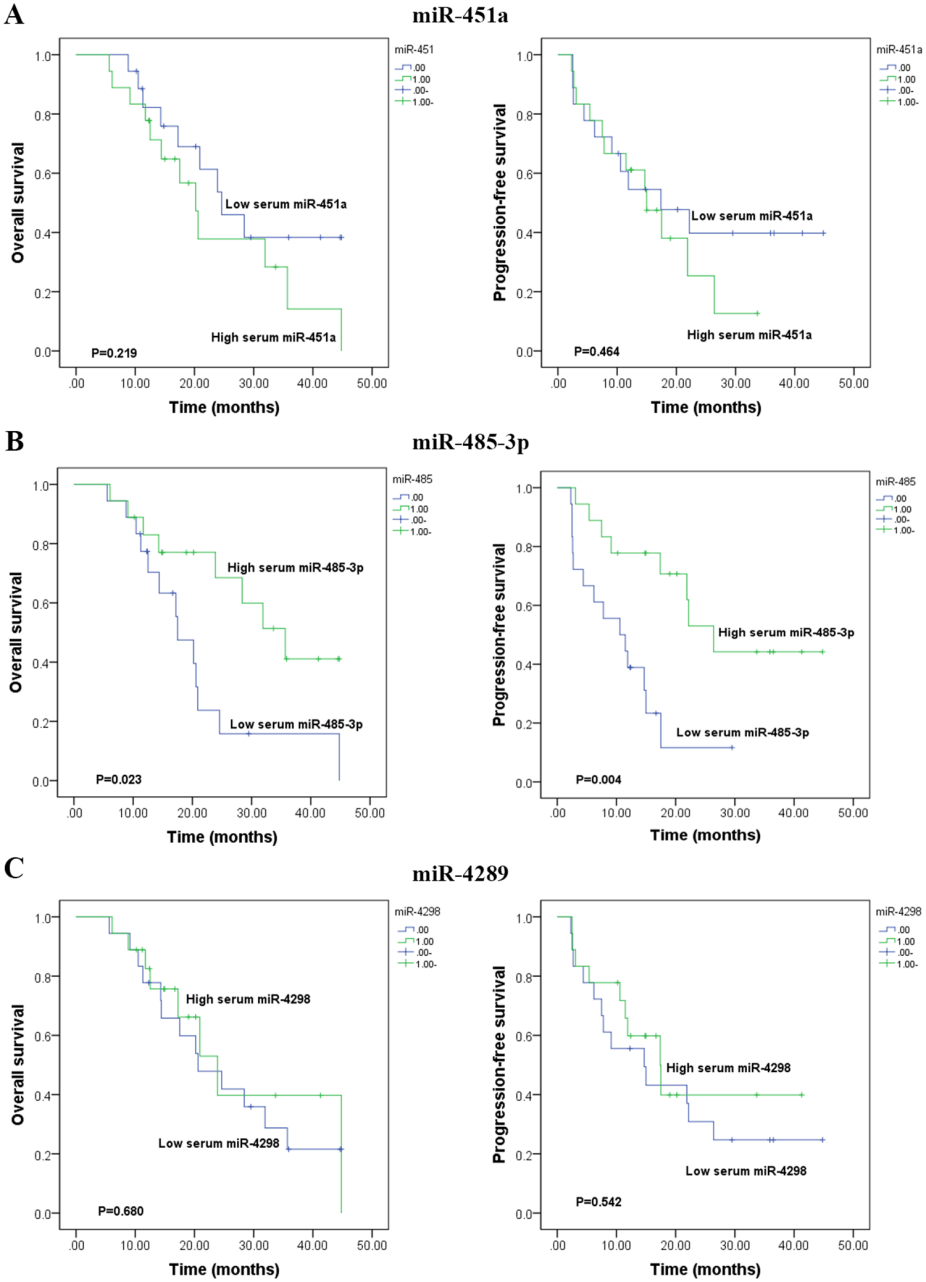
Comparison of overall survival (OS) and progression-free survival (PFS) of the GBM patients with lowand high levels of serum miR-451a, miR-485-3p and miR-4298. **(A)** There is no difference of survivals (OS and PFS) between patients with a low and high level of serum miR-451a **(B)** Patients with a low level of serum miR-485-3p had significantly shorter OS (P = 0.023) and PFS (P = 0.004) than those with a high level. **(C)** There is no difference of survivals (OS and PFS) between patients with a low and high level of serum miR-4298.

To evaluate the prognostic significance of the serum miRNAs in GBM patients, the three miRNAs were analyzed by Kaplan-Meier and Log-rank models. It was demonstrated that patients with a low expression of miR-485-3p had significantly shorter PFS and significantly worse OS in GBM patients (n = 36) (P = 0.004 and P = 0.023, Fig 2B). No significant correlation was found between survival rates (PFS and OS) and the expression levels of miR-451a and miR-4298 in GBM patients (both P >0.05, Fig 2A and 2C).

To determine whether serum miR-485-3p expression level was an independent risk factor for prognosis of GBM patients, the Cox univariate and multivariate hazard regression models were employed to analyze the miRNAs and clinical features. As shown in Tables 2 and 3, serum miR-485-3p expression level was an independent factor for predicting the PFS (RR: 0.135, 95% CI: 0.048–0.382, P <0.001, Table 2) and OS (RR: 0.171, 95% CI: 0.056–0.523, P = 0.002, Table 3) in GBM patients. Other independent prognostic factors for PFS and OS include age, preoperative Karnofsky Performance Scale (KPS) and surgery procedure (Tables 2 and 3).

**Table 2:** Univariate and multivariate COX proportional hazard analyses of prognostic factors for progression-free survival in GBM patients.

Variables	Univariate analysis		Multivariate analysis	
	RR (95% CI)	P value	RR (95% CI)	P value
Gender				
Male vs Female	0.865 (0.369–2.028)	0.739		
Age (years)				
≥45 vs < 45	1.036 (1.001–1.072)	0.047	6.704 (2.009–22.370)	0.002
Preoperative KPS				
80–100 vs < 80	0.345 (0.134–0.890)	0.028	0.267 (0.094–0.755)	0.013
Surgery procedure				
PR vs GTR	2.685 (1.045–6.898)	0.040	5.364 (1.872–15.366)	0.002
Tumor size (cm)				
≥5 vs < 5	1.286 (0.547–3.026)	0.564		
Tumor MGMT expression				
Pos vs Neg	2.162 (0.870–5.370)	0.097		
Serum miR-451a level				
High vs Low	1.372 (0.586–3.214)	0.466		
Serum miR-485-3p level				
High vs Low	0.276 (0.110–0.690)	0.006	0.135 (0.048–0.382)	<0.001
Serum miR-4298 level				
High vs Low	0.756 (0.319–1.793)	0.526		

Abbreviations: RR, risk ratio; 95% CI, 95% confidence interval; KPS, Karnofsky Performance Scale; GTR, gross total resection; PR, partial resection; MGMT, O(6)-methylguanine-DNA methyltransferase detected by immunohistochemistry; Pos, positive; Neg, Negative.

**Table 3:** Univariate and multivariate COX proportional hazard analyses of prognostic factors for overall survival in GBM patients.

Variables	Univariate analysis		Multivariate analysis	
	RR (95% CI)	P Value	RR (95% CI)	P Value
Gender				
Male vs Female	0.924 (0.388–2.202)	0.858		
Age (years)				
≥45 vs < 45	1.036 (1.000–1.072)	0.049	7.708 (1.952–30.437)	0.004
Preoperative KPS				
80–100 vs < 80	0.828 (0.757–0.905)	<0.001	0.111 (0.031–0.401)	0.001
Surgery procedure				
PR vs GTR	3.027 (1.082–8.464)	0.035	6.939 (2.070–23.258)	0.002
Tumor size (cm)				
≥5 vs < 5	1.424 (0.588–3.448)	0.433		
Tumor MGMT expression				
Pos vs Neg	1.903 (0.763–4.747)	0.168		
Serum miR-451a level				
High vs Low	1.678 (0.619–4.551)	0.309		
Serum miR-485-3p level				
High vs Low	0.360 (0.144–0.898)	0.029	0.171 (0.056–0.523)	0.002
Serum miR-4298 level				
High vs Low	0.829 (0.340–2.020)	0.681		

Abbreviations: RR, risk ratio; 95% CI, 95% confidence interval; KPS, Karnofsky Performance Scale; GTR, gross total resection; PR, partial resection; MGMT, O(6)-methylguanine-DNA methyltransferase detected by immunohistochemistry; Pos, positive; Neg, Negative.

## Discussion

GBM is the most lethal neurological malignancy with a very poor prognosis [2]. Thus, it is a major clinical challenge to identify sensitive and specific biomarkers for the prognosis of this malignancy, as well as to develop new molecularly targeted therapeutic agents for this lethal disease. Multiple studies have explored miRNA as biomarkers in GBM based on the deregulation of miRNA expression in tumor tissue [28–30]. However, tissue-specific miRNAs can be detected only in tumor tissues, which limits their potential application in clinical practice. Circulating biomarkers, such as miRNAs in serum or plasma, can be measured noninvasively and would be easily testable. Therefore, increasing efforts have been made in developing tumor biomarkers in blood, serum or plasma. In 2011, Roth et al profiled miRNA expression of peripheral blood mononuclear cells in 20 GBM patients and matched healthy volunteers with biochip containing 1158 human mature miRNAs, and found miR-128, miR-342-3p and a signature (consisting of 180 miRNAs) could be used to distinguish patients and healthy volunteers [16]. Interestingly, they also found that miR-485-3p was decreased in GBM patients, which is concordant with our result. In 2012, Wang et al reported 3 serum miRNAs measured by qRT-PCR could be used as diagnostic markers for glioma including GBM [17]. In 2013, Yang et al identified a serum seven-miRNA signature to diagnose malignant astrocytoma [18]. Recently, Tang and Yue groups discovered that plasma/serum miR-185 and miR-205 could be potential biomarkers for diagnosis and/or prognosis in human glioma including GBM [31, 32]. However, most of these studies are focused on single miRNAs, which is not derived from high-throughput screening experiments on a suitable GBM sample size and may not be the best biomarkers for diagnosis and/or prognosis of GBM patients.

In a previous study, we made a genome-wide profiling of miRNA expression in the serum in 22 GBM patients and 8 healthy volunteers with a custom microarray containing 1849 miRNAs and identified 19 differentially expressed miRNAs between patients and healthy controls (fold change>1.5) [19]. In the present study, based on the profiling result of serum miRNAs in GBM patients, we first carried out qRT-PCR on 3 miRNAs (miR-451a, miR-485-3p and miR-4298) in an new cohort (24 GBM patients and 12 healthy volunteers) to validate the microarray data. The results show that the expression levels of the 3 miRNAs detected by qRT-PCR are significantly different between GBM patients and healthy volunteers, which are consistent with the microarray data. Then, we detected the 3 serum miRNA expressions with qRT-PCR in another 12 GBM patients, and analyzed their clinical significance in all 36 GBM patients. The result shows that serum miR-485-3p is significantly reduced in GBM patients than in healthy volunteer and associated with PFS and OS of GBM patients. Multivariate Cox regression analysis demonstrates that serum miR-485-3p is an independent prognostic factor for PFS and OS in GBM patients, which suggests that miR-485-3p may be involved in GBM development and prognosis, and functions as a tumor suppressor.

miR-485-3p, together with miR-154, miR-299-5p, miR-376a, etc, is mapped to the 14q32.31 region in which allelic deletions [33] and translocations [34] are frequently identified in cancer, suggesting that miR-485-3p may be a tumor suppressor. Lou et al reported that miR-485-3p is decreased in breast cancer tissues and can inhibit migration and invasion of breast cancer cells by targeting PGC-1α [35]. In 2011, Chen et al. provided evidence that increased levels of miR-485-3p can decrease the chemoresistance of human leukemic lymphoblastic cells to Teniposide by targeting NFYB gene [26]. In sharp contrast, Lucotti et al found that miR-485-3p increased the chemoresistance of DU-145 prostate cancer cells to Fludarabine by targeting NFYB [27], implying that miR-485-3p may have different functions in different conditions. In addition, miR-485-3p may promote proliferation, migration/invasion or apoptosis by targeting NTRK3 or MAT1A in HCC cells [36, 37]. These studies demonstrate that miR-485-3p has different functions in different cancers.

In conclusion, we have demonstrated for the first time that serum miR-485-3p is a potential prognostic marker for GBM patients. In future research, we will investigate the roles of miR-485-3p in GBM pathogenesis and analyze its relevant molecular mechanisms.
